# The common link between sleep apnea syndrome and osteoarthritis: a literature review

**DOI:** 10.3389/fmed.2024.1401309

**Published:** 2024-08-21

**Authors:** Lian Weng, Yuxi Luo, Xiongjunjie Luo, Kaitao Yao, Qian Zhang, Junjie Tan, Yiran Yin

**Affiliations:** ^1^Luzhou Longmatan District People's Hospital, Luzhou, China; ^2^Department of Orthopedics, The Affiliated Hospital of Southwest Medical University, Luzhou, China; ^3^Sichuan Provincial Laboratory of Orthopedic Engineering, Luzhou, China; ^4^Department of Clinical Medicine, Southwest Medical University, Luzhou, China

**Keywords:** sleep apnea syndrome, osteoarthritis, obesity, age, comorbidity

## Abstract

Patients with Osteoarthritis (OA) often also suffer from Sleep Apnea Syndrome (SAS), and many scholars have started to notice this link, although the relationship between the two is still unclear. In this review, we aim to summarize the current literature on these two diseases, integrate evidence of the OA and OSA connection, explore and discuss their potential common mechanisms, and thus identify effective treatment methods for patients with both OA and SAS. Some shared characteristics of the two conditions have been identified, notably aging and obesity as mutual risk factors. Both diseases are associated with various biological processes or molecular pathways, including mitochondrial dysfunction, reactive oxygen species production, the NF-kB pathway, HIF, IL-6, and IL-8. SAS serves as a risk factor for OA, and conversely, OA may influence the progression of SAS. The effects of OA on SAS are underreported in the literature and require more investigation. To effectively manage these patients, timely intervention for SAS is necessary while treating OA, with weight reduction being a primary requirement, alongside combined treatments such as Continuous positive airway pressure (CPAP) and medications. Additionally, numerous studies in drug development are now aimed at inhibiting or clearing certain molecular pathways, including ROS, NF-KB, IL-6, and IL-8. Improving mitochondrial function might represent a viable new strategy, with further research into mitochondrial updates or transplants being essential.

## Background

Sleep apnea syndrome (SAS) is a breathing-related disorder during sleep, where breathing frequently pauses or becomes significantly shallow. These pause events may last 10 s or longer and occur more than 5 times per hour. Recent studies show that approximately half of the global population is affected by SAS (1). Research investigating the Asian obese population has discovered a SAS prevalence rate of up to 80.5% (2). Osteoarthritis (OA), a chronic degenerative disease occurring in the joints, has been seeing a rise in prevalence with the aging of the population.

A close relationship exists between SAS and OA. In a cross-sectional study, Asiye Kanbay et al. (3) identified SAS as a risk factor for OA. Furthermore, Andressa Silva et al. (4) observed that patients diagnosed with both OA and SAS exhibited poorer knee joint function than those without SAS. Present evidence suggests the importance of focusing on the connection between OA and SAS. Hence, this review aims to summarize the shared aspects between the two, providing directions for further research into their comorbid issues.

## Method

Searches were performed in PubMed and Google Scholar using keywords like “sleep apnea syndrome,” “osteoarthritis,” “obstructive sleep apnea,” and terms such as “arthritis,” “arthropathy,” “joint pain.” By continuously recombining search terms and filtering through titles and abstracts, we identified relevant documents concerning the relationship between SAS and OA. Using the snowballing method, we summarized the content of retrieved literature and identified additional search terms related to OA and SAS, which facilitated further searches. Inclusion criteria included that the full text be in English and published in this century. Excluded were those studies that did not meet the objectives of this review and were unrelated to the topic.

## Common risk factors for SAS and OA

### Obesity

Obesity is a risk factor for SAS. Compared to individuals of normal weight, those who are obese or overweight are significantly more likely to suffer from SAS, and they tend to experience a higher severity of the condition. As the Body Mass Index (BMI) and the circumference of the neck and waist increase, the incidence of SAS escalates, and sleep quality deteriorates significantly (5–7).

Bariatric surgery demonstrates remarkable outcomes in treating obstructive sleep apnea in obese patients, particularly in cases of morbid obesity. These surgeries are effective in not only reducing apnea-hypopnea events but also in decreasing body weight, and improving oxygen saturation, deep N3 sleep, and REM (Rapid Eye Movement) sleep stages. Moreover, the surgery improves pulmonary function by increasing Forced Vital Capacity (FVC) (8). Additionally, research shows that managing obesity-related body metrics through pharmacological intervention can reduce symptoms of sleep apnea (9). Wyszomirski et al. (10) discovered that sleep apnea can exacerbate obesity. However, research by Caliendo et al. (11) suggests there may not be a direct correlation between obesity and sleep apnea, yet a positive relationship exists between BMI and the severity of AHI. In summary, obesity is a risk factor for SAS, and managing obesity can play a key role in the prevention and control of sleep apnea.

Obesity is closely related to OA. Research shows that both overall and abdominal obesity elevate the risk of knee OA, with the risk peaking when these two types of obesity are present simultaneously (12). In South Korea’s aged population, obesity significantly raises the risk of OA (13). Research in Saudi Arabia has discovered a close relationship between obesity and bilateral knee OA (14). Within an OA animal model, researchers induced obesity in mice with a high-fat diet, thereby speeding up the mice’s OA progression (15). Further research indicates a causal link between obesity and hip OA (16). Interestingly, there is an association between metabolic syndrome and hip OA in women, but in men, this link is not evident. However, for men, obesity is directly associated with hip OA, yet in women, this association is less clear. The mechanical impact of obesity may be the main mechanism behind hip OA in men, while in women, metabolic actions are believed to take precedence (17). Not only do obesity and metabolic syndrome foster knee OA through increased mechanical loading, but they also do so via systemic inflammation caused by obesity (18). Moreover, obesity could intensify OA pain, with class III obese patients suffering from notably greater pain than those who are overweight or have class I or II obesity (19).

OA may in turn worsen obesity. Research indicates a higher incidence of obesity, sarcopenia, and sarcopenic obesity in patients with end-stage knee OA. Patients with bilateral knee OA exhibit a higher rate of sarcopenic obesity compared to those with unilateral knee OA (20), underscoring the significance of focusing on the interplay between obesity and OA. Interestingly, bariatric surgery can alleviate symptoms of OA and even potentially slow its progression (21).

### Age

Age has an impact on SAS. With advancing age, the severity of SAS tends to intensify, particularly among men (22). For women, transitioning into menopause significantly raises the risk of developing SAS (23). The prevalence of SAS in elderly women is on the rise, with this increase possibly due to aging causing a decrease in the reactivity of upper airway muscle activity, thereby making the airway more prone to collapse (24).

SAS may accelerate the aging process, acting as a contributing factor to aging-related conditions (25). SAS is associated with aging characteristics such as metabolic syndrome, vascular dysfunction, decline in quality of life and cognitive scores, accelerated brain aging, increased insulin resistance, and elevated levels of plasma hydrogen peroxide, GSH, IL-6, hsCRP, and leptin (26). For those under 50, sleep apnea patients are particularly susceptible to aging-related mechanisms such as altered cell communication, impaired nutrient sensing, telomere shortening, mitochondrial dysfunction, and genomic instability (26). SAS can affect the cognition of the elderly. Research indicates that obstructive sleep apnea is linked to a decline in cognitive abilities, particularly in attention and processing speed, among middle-aged and older people (27, 28). This could be attributed to minor alterations in the local brain structures of individuals with SAS (29).

Age significantly impacts OA. Research analyzing data from 36 Organization for Economic Cooperation and Development countries (OECD) countries identified aging and obesity as critical risk factors for OA (30). Both obesity and age are closely related to the severity of knee OA, with age being an independent risk factor for its severity (31). In studies on aged female mice, increased knee cartilage degeneration was observed, alongside more severe mechanical allodynia, knee joint hyperalgesia, and decreased grip strength (32). Di et al. (33) observed that the incidence of knee OA increased most significantly in the age groups of 35 to 39 and 40 to 44 years. A meta-analysis highlighted age as a risk factor for scapulothoracic OA (34). Furthermore, age also influences the hand strength in patients with hand OA (35).

## Common mechanism of SAS and OA

### Mitochondrial dysfunction

Mitochondrial dysfunction plays a crucial role in the context of SAS. A diet high in fats causes mitochondrial dysfunction and oxidative stress, which damages the genioglossus muscle and could lead to SAS (36). Moreover, research has shown that in the whole blood DNA of patients with SAS, the copy number of mitochondrial DNA (mtDNA) is reduced, with this reduction being associated with the severity of the patient’s condition (37). However, Lacedonia D et al. found that: in patients, intermittent hypoxia intensifies oxidative stress, resulting in mitochondrial damage, and consequently, a increase in the number of mitochondrial DNA copies (38).

Chronic intermittent hypoxia in patients can lead to mitochondrial dysfunction, triggering neuroinflammation, an increase in reactive oxygen species (ROS) (39). Furthermore, mitochondrial dysfunction is associated with metabolic disorders, and the level of mitochondrial-derived peptide MOTS-c in serum is closely related to SAS. MOTS-c emerges as a new and useful marker for early metabolic disorder in patients (40). Interestingly, enhancing mitochondrial autophagy to remove damaged mitochondria can alleviate tissue cell damage caused by the syndrome. Research has found that silencing NLRP3 in mice activates the PINK1-Parkin pathway to promote mitochondrial autophagy, thereby preventing the neuroinflammation and production of reactive oxygen species induced by intermittent hypoxia (41).

Mitochondrial dysfunction is indeed a significant factor contributing to the development of OA. Cytokine stimulation triggers mitochondrial dysfunction through signaling pathways such as JNK, PI3K/Akt, NF-κB, and p38 MAPK, which is characterized by decreased transmembrane potential, reduced ATP production, and increased ROS, all of which contribute to cartilage cell damage (42). The ensuing ROS chain reaction caused by mitochondrial dysfunction damages mitochondrial DNA (mtDNA) and cardiolipin, leading to a decrease in mitochondrial membrane potential and the opening of mitochondrial permeability transition pores (PTP). This facilitates the influx of calcium ions (Ca2+) and thus triggers an inflammatory response. During this process, the inflammasome NLRP3 is activated, enabling the conversion from pro-Caspase-1 to Caspase-1 and initiating a caspase cascade, ultimately leading to pyroptosis. Moreover, ROS can further activate NF-κB, resulting in changes in gene transcription that lead to cell cycle arrest, cellular senescence, and degradation of the extracellular matrix (43).

### Reactive oxygen species (ROS)

Intermittent hypoxia caused by SAS leads to the activation of NADPH oxidase and xanthine oxidase, mitochondrial dysfunction, and uncoupling of nitric oxide synthase (NOS) (44, 45). These reactions result in increased ROS levels, further activating transcription factors such as NF-kB and AP-1, which then trigger the release of inflammatory mediators and pro-inflammatory cytokines (45, 46), ultimately causing tissue and cellular damage (Table 1).

**Table 1:** The role of ROS in SAS and OA.

Impact/Drug	Molecule/pathway	Function	
Hypoxia	mtROS(+)	Activation of the NLRP3 inflammasome	Wu et al. (41)
Hypoxia	ROS(+)，NF-κB(+)，HIF-1α(+)	Pyroptosis of muscle cells	Yu et al. (47)
Hypoxia	ROS(+)，NF-kB(+)，AP-1(+)	Promotes the release of inflammatory mediators and proinflammatory cytokines	Lavie (45)
Synovial inflammation	ROS/NLRP3(+)	Chondrocyte apoptosis accelerates cartilage degeneration in osteoarthritis	Liu et al. (48)
Nitidine chloride	ROS(−)	Reduces inflammation and cellular senescence in mice with osteoarthritis	Lin et al. (49)
Mn(3)O (4)/UIO-TPP nano-enzyme	ROS(−)	Restore mitochondrial function and relieve osteoarthritis	Zhang et al. (50)
Fucoxanthin	ROS(−)	It plays a protective role in chondrocytes and effectively reduces the development of osteoarthritis	Wu et al. (51)
Polydopamine-Pd nanozymes	ROS(−)	Anti-oxidant and anti-inflammatory	Hu et al. (52)
Nodakenin	Drp1/ROS/NLRP3 (−)	Reduce cartilage degradation and inflammation	Yi et al. (53)
TRPV4(−)	ROS/NLRP3 (−)	M1 macrophage polarization was hindered and osteoarthritis was delayed	Sun et al. (54)
NP@Poly(RHAPM)	ROS(−)，Macrophage repolarization (+)	Repolarization of M1 macrophages to the M2 phenotype enhanced chondrocyte proliferation and viability	Li et al. (55)
PIM-1(−)	ROS/Cl(−)(−)	Inhibition of NLRP3 inflammasome activation can alleviate osteoarthritis	Zhang et al. (56)
Pt@PCN222-Mn cascade nanase	ROS-NF-κB(−)，MAPK (−)	It also inhibited the production of inflammatory factors, ECM degradation and chondrocyte apoptosis	Zhang et al. (57)
Dendrobine	ROS/NF-κB (−)	Reduce osteoarthritis	Chen et al. (58)
Astragalus polysaccharide	ASK1/p38 MAPK(−)	Thioredoxin is activated and apoptosis is inhibited	Xu et al. (59)
++GLX351322(GLX)	NOX4(−)，ROS(−)	Inhibition of inflammatory responses	Zhen et al. (60) and Huang et al. (61)
Angelica Polysaccharide	PPARγ(+)，SOD2(+)，ROS(−)	Improve mitochondrial metabolism of chondrocytes in osteoarthritis	Ni et al. (62)

* “+” represents facilitation, “−” represents inhibition.

Additionally, intermittent hypoxic conditions during apnea lead to an increase in mtROS and activation of NLRP3 inflammasomes, these inflammatory reactions may cause damage to other organs (41). Hypoxia-induced increase in ROS also promotes cell apoptosis through the activation of the NF-κB/HIF-1α signaling pathway (47).

ROS plays a crucial role in the development mechanism of OA (Table 1). Research indicates that synovitis-induced activation of the ROS/NLRP3 pathway can enhance chondrocyte apoptosis, thereby speeding up the degeneration of joint cartilage in OA (48). Efficiently clearing ROS can alleviate the symptoms of OA. For instance, nitidine chloride, acting as a reactive oxygen species scavenger, can reduce inflammatory responses and cell senescence in a mouse model of OA (49). Moreover, mitochondria-targeted Mn(3)O (4)/UIO-TPP nanozymes restore mitochondrial function by eliminating ROS, thus effectively combating OA (50). Molybdenum-based polyoxometalate (POM) nanoclusters leverage their near-infrared photosensitivity to enhance ROS scavenging capability, markedly improving clinical symptoms of OA (63). Additionally, Nodakenin can mitigate cartilage degeneration and inflammatory responses in a mouse model of OA by modulating the mitochondrial Drp1/ROS/NLRP3 axis (53).

The ROS/NLRP3 pathway also impedes M1 macrophage polarization, leading to OA. By inhibiting the ROS/NLRP3 pathway, the progression of OA can be delayed (54). Researchers have created a nanomaterial NP@Poly(RHAPM) that is capable of significantly lowering ROS levels within cells, thereby leading to the repolarization of M1 macrophages to the M2 phenotype, enhancing the proliferation and vitality of chondrocytes, as well as preventing cell apoptosis (55). Similarly, BTZ@PTK reduces ROS levels, activates apoptosis in M1 macrophages, inhibits M1 macrophage-mediated inflammatory responses, and thus improves OA (64).

ROS regulate the NF-κB and MAPK pathways, thus contributing to OA. The Pt@PCN222-Mn cascaded nanoenzyme, developed by Zhang et al. (57), can delay the progression of temporomandibular joint OA in rat models by inhibiting the ROS-NF-κB and MAPK signaling pathways. Moreover, Dendrobine targets chondrocytes via the ROS/NF-κB axis, inhibiting the expression of aging-related secretory phenotype factors, thus helping to alleviate OA (58). Additionally, Astragalus polysaccharides inhibit apoptosis and improve OA symptoms by suppressing ROS-mediated activation of the ASK1/p38 MAPK signaling pathway, subsequently activating thioredoxins (59).

### NF-κB pathway

Chronic hypoxia mediated by SAS can cause damage to multiple organ tissues throughout the body via the NF-κB pathway (Table 2). Hypoxia-induced ROS promotes myoblast apoptosis in obstructive sleep apnea via the NF-κB/HIF-1α signaling pathway (47). Additionally, hypoxia accelerates lung fibrosis in mouse lung injury by regulating the NF-κB/Nrf2 signaling pathway (65). Hypoxia further mediates adipocyte insulin resistance through the RAGE/NF-κB pathway (66). The activation of the TLR4/NF-κB/VEGF pathway enhances vascular dysfunction in the soft palate, aggravating SAS (67).

**Table 2:** The role of NF-κB pathway in SAS and OA.

Impact/Drug	Molecule/pathway	Function	
Hypoxia	NF-κB(+), Nrf2(−)	Pulmonary fibrosis that accelerates lung injury in mice	Kang et al. (65)
Hypoxia	RAGE(+), NF-κB (+)	Increased insulin resistance in adipocytes	Tang et al. (66)
HMGB1(+)	TLR4/NF-κB/VEGF(+)	Promote soft palate vascular dysfunction in patients with obstructive sleep apnea	Su et al. (67)
NF-κ b(+)	JNK(+)	Hippocampal neuronal apoptosis and cognitive dysfunction	Liu et al. (68)
PDTC, Rapa	mTOR (−), NF-κB (−)	Prevention of hypoxia-induced hippocampal neuronal damage	Zhang et al. (69)
o-glcn acylation	NFAT(−), NF-κB(−)	Attenuated intermittent hypoxia-induced cardiac remodeling	Nakagawa et al. (70)
miR-15b-5p/miR-92b-3p(−)	PTGS1-NF-κB-SP1(+)	Oxidative stress and MAOA hyperactivation	Chen et al. (71)
Indole-3-propionic acid	AhR/NF-κB (−)	Reduces chondrocyte inflammation and osteoarthritis	Zhuang et al. (72)
Phillygenin	Nrf2(+), NF-κB (−)	Inhibition of chondrocyte inflammation	Zhang et al. (73)
Orientin	Nrf2/HO-1(+), SIRT6 pathway(+), NF-κB (−)	Inhibits the development of osteoarthritis	Xia et al. (74)
Pachymic acid	Sirtuin 6(+), NF-κB(−)	It can inhibit the inflammatory response of chondrocytes and alleviate the progression of osteoarthritis	Wu et al. (75)
Pelagonidin	NF-κB(−)	Improve inflammation and cartilage degeneration in osteoarthritis	Zeng et al. (76)
Sakuranetin	PI3K/AKT/NF-κB (−)	Reduces inflammation and chondrocyte dysfunction in osteoarthritis	Deng et al. (77)
miR-4738-3p	NF-kB(−),COL1A2(−)	Relieve the symptoms of low-grade inflammation in osteoarthritis	Xu et al. (78)
Dendrobine	ROS/NF-κB(−)	Alleviates cellular senescence and osteoarthritis	Chen et al. (58)
Oxymatrine	Nrf2(+), NF-κB(−)	Reduced proinflammatory cytokines, ECM degradation and OA progression	Zhou et al. (79)
Chrysophanol	Sirt6(+), Nrf2(+), NF-κB(−)	Alleviates inflammation and ECM degradation in osteoarthritis	Lu et al. (80)
Rutaecarpine	PI3K/AKT/NF—κB(−), MAPK(−)	Improving osteoarthritis	Wan et al. (81)
Mulberroside A	MAPK(−), NF-κB(−), PI3K-AKT–mTOR (−)	Relieving Osteoarthritis	Lu et al. (82)
Ergothioneine	Sirt6(+), NF-κB(−)	Inhibit the progression of osteoarthritis	Wang et al. (83)
Plantamajoside	NF-κB(−), MAPK(−)	Relieving Osteoarthritis	Lin et al. (84)
Paroxetine	NF-κB (−)	Reduce chondrocyte apoptosis and inhibit osteoclast formation	Zheng et al. (85)
Stevioside	NF-κB(−), MAPK (−)	Inhibition of chondrocyte inflammation and apoptosis	Cai et al. (86)
Forkhead box O3	NF-κB(−), MAPK(−)	Inhibition of ferroptosis in chondrocytes	Zhao et al. (87)
Suramin	Nrf2/HO-1(+), NF-κB (−)	Improving osteoarthritis	Shen et al. (88)
κ-opioid receptor	NF-κB (−)	Promote macrophage polarization and reduce osteoarthritis synovitis	Shi et al. (89)
miR-181a-5p	DDX3X(−), NF-ΚB(−)	Inhibit the progression of osteoarthritis	Zhao et al. (90)
MiR-203a-3p	MYD88/NF-κB (−)	Reduce the progression of osteoarthritis	Chen et al. (91)
Mechanical stress	tgf—β 1/Smad2/Smad3 axis(+), NF-ΚB(−)	Prevent pyroptosis of chondrocytes	Wang et al. (92)
Non-weight bearing exercise	TLR4/MyD88/NF-κB (−)	It can reduce the levels of inflammatory cytokines il-1β, IL-6 and tnf-α, and slow down the degeneration of articular cartilage	Wang et al. (93)
Regulating tendons and bone-setting techniques	TLR4-MyD88-NF-κB (−)	Reduce synovial inflammation of the knee joint	Jin et al. (94)
Moderate exercise	metrnl(+), PI3K/Akt/NF-κB(−), NLRP3/caspase-1/GSDMD (−)	Improves inflammation and pyroptosis	Liu et al. (95)
ROR1(+)	STAT3(+), NF-κB (+)	There was an imbalance between anabolism and catabolism in chondrocytes	Huang et al. (96)
SNIP1(+)	NF-κB(−)	Reduces extracellular matrix degradation and inflammation	Chen et al. (97)
FTO(+)	TLR4/MyD88/NF-κB(−)	Relieve osteoarthritis	Cai et al. (98)

* “+” represents facilitation, “-” represents inhibition.

Intervening in the NF-κB pathway offers a therapeutic avenue for neurological and cardiovascular diseases caused by SAS. Liu et al. (68) demonstrated that inhibiting NF-κB activation can alleviate cognitive impairment in sleep apnea. Similarly, PDTC and Rapa, by inhibiting the mTOR and NF-κB pathways, can prevent damage to hippocampal neurons induced by hypoxia (69). Song et al. (99) showed that inhibiting endothelial NF-κB signaling can alleviate arteriosclerosis induced by chronic intermittent hypoxia in mice. Furthermore, enhanced O-GlcNAcylation can inhibit NFAT and NF-κB activity in mice to attenuate cardiac remodeling induced by intermittent hypoxia (70). Also, overexpressing miR-15b-5p/miR-92b-3p and inhibiting the PTGS1-NF-κB-SP1 signaling pathway provides a potential treatment for depression related to SAS (71).

There’s a significant link between the NF-κB pathway and OA (Table 2); suppressing NF-κB can significantly mitigate OA. Pelagonidin effectively improves OA by inhibiting the NF-κB pathway, thus reducing inflammation and cartilage degeneration (76). Exosomal miR-4738-3p helps relieve mild inflammatory symptoms of OA by regulating COL1A2 through the NF-kB and inflammatory signaling pathways (78). Atractylenolide III effectively alleviates OA and chondrocyte aging by inhibiting the NF-κB signaling pathway (100). Shihuakalin targets cellular aging and OA via the ROS/NF-κB pathway (58). Quercitrin acts to slow down OA by inhibiting the NF-κB signaling pathway and enhancing glucose transport capability (101). Indole-3-propionic acid offers relief to chondrocyte inflammation and OA through the AhR/NF-κB axis (72).

Furthermore, NF-κB is known to promote chondrocyte apoptosis, and inhibiting NF-κB can effectively protect chondrocytes. Paroxetine has been shown to alleviate chondrocyte apoptosis and inhibit osteoclast formation by suppressing NF-κB (85). Stevioside reduces chondrocyte inflammation and apoptosis *in vivo* via the NF-κB and MAPK pathways, thereby improving OA (86). Forkhead box O3 plays a crucial role in inhibiting chondrocyte ferroptosis and alleviating OA by suppressing the NF-κB/MAPK signaling (87).

NF-κB plays a pivotal role in OA through its influence on macrophages. Suramin effectively ameliorates OA by targeting the Nrf2/HO-1 and NF-κB signaling pathways in chondrocytes and by promoting the M2 polarization of macrophages (88). Additionally, κ-opioid receptor activation can modulate macrophage polarization through the NF-κB pathway and help alleviate osteoarthritic synovitis (89).

Some miRNAs significantly influence OA through the NF-κB pathway. The MicroRNA-15a/β1, 4-galt-i axis is involved in the degeneration of osteoarthritic cartilage via the NF-κB signaling pathway (102). MiR-203a-3p plays a role in alleviating chondrocyte apoptosis by regulating the MYD88/NF-κB pathway (91).

Physical factors also impact OA via the NF-κB pathway. Mechanical stress works to avert chondrocyte apoptosis by suppressing the NF-κB signaling pathway (92). Non-weight-bearing exercise has been shown to reduce rat knee OA through the TLR4/MyD88/NF-κB signaling pathway (93). Tendon adjustments and bone grafting are effective in facilitating the healing of synovitis in rabbit osteoarthritic knees via the TLR4-MyD88-NF-κB pathway (94). Moderate-intensity physical activity can mitigate inflammation and cellular apoptosis by enhancing metrnl release, thereby acting to suppress the PI3K/Akt/NF-κB and NLRP3/caspase-1/GSDMD pathways (95).

Upregulating Nrf2 to inhibit NF-κB activity offers a promising approach to alleviate OA. Li et al. (103) found that the Nrf2/HMGB1/NF-κB axis regulates chondrocyte apoptosis and extracellular matrix degradation in OA. Phillygenin improves OA in mice by inhibiting chondrocyte inflammation via the Nrf2/NF-κB axis (73). Oxymatrine enhances OA treatment *in vitro* and *in vivo* via the Nrf2/NF-κB axis (79). Chrysophanol acts to prevent OA inflammation and extracellular matrix degradation via the Nrf2/NF-κB axis (80).

The Sirtuin 6/NF-κB signaling pathway holds significance in OA treatment. Orientin effectively mitigates OA through activation of the SIRT6 signaling pathway and suppression of the NF-κB pathway (74). Pachymic acid targets chondrocyte inflammation by modulating the Sirtuin 6/NF-κB signaling axis (75). Emodin again demonstrates its efficacy in preventing OA inflammation and extracellular matrix degradation in chondrocytes via the Sirt6/NF-κB pathway (80). Ergothioneine is proven to inhibit the progression of OA both *in vitro* and *in vivo* via the Sirt6/NF-κB axis (83).

The PI3K/AKT/NF-κB and MAPK signaling pathways are closely associated with the onset and progression of OA. Sakuranetin acts to reduce inflammation and chondrocyte dysfunction in OA by targeting the PI3K/AKT/NF-κB pathway (77). Rutaecarpine demonstrates effectiveness in improving OA by inhibiting PI3K/AKT/NF-κB and MAPK signal transduction through integrin αVβ3 (81). Mulberroside A contributes to alleviating OA by restoring damaged autophagy and inhibiting the MAPK/NF-κB/PI3K-AKT–mTOR signaling pathway (82). Furthermore, Plantamajoside beneficially impacts the development of OA by suppressing the activation of NF-κB and MAPK (84).

### HIF (hypoxia-inducible factors)

HIF-1α is recognized as a biomarker for SAS (104). In patients with sleep apnea, the HIF-1α protein expression levels are notably elevated, correlating with the chronic hypoxia state (105). This chronic hypoxia not only leads to other diseases, through HIF-1 (106) but also results in an increase in ROS, which further promotes tissue cells apoptosis via the NF-κB/HIF-1α signaling pathway (47). Moreover, elevated HIF-1α levels are tied to excessive expression of circadian rhythm proteins, thereby increasing the risk of circadian rhythm disturbances in SAS patients (107). SAS may harm other tissues and organs via HIF-1, SAS can accelerate the progression of aortic dissection through the ROS-HIF-1α-MMPs related pathway (108), as well as exacerbate neuroinflammation and apoptosis in early brain injury after subarachnoid hemorrhage via the ASC/HIF-1α pathway (109).

HIF-2α is a subtype of the HIF. HIF-2α increases the expression of Superoxide Dismutase 2 (SOD2), an antioxidant enzyme that can reduce ROS (110). Chronic hypoxia from SAS causes an increase in ROS, subsequently triggering a rise in intracellular calcium levels ([Ca(2+)]i), which stimulates increased synthesis of HIF-1α and enhanced degradation of HIF-2α, as a result, the normal balance between HIF-1α-dependent pro-oxidants and HIF-2α-dependent antioxidants is broken, resulting in an additional increase in ROS, this exacerbates damage to multiple tissues throughout the body (111).

Some studies indicate that HIF-1 plays a protective role in OA (Table 3). HIF-1α may protect articular cartilage by promoting chondrocyte phenotype, maintaining chondrocyte vitality, and supporting metabolic adaptation in hypoxic conditions (128). In hypoxic conditions, the activity of HIF-1α is enhanced, and it accelerates angiogenesis in cartilage through the vascular endothelial growth factor VEGF and Notch signaling pathway (112). Sunli Hu et al. (129) found that HIF-1α-mediated mitochondrial autophagy can alleviate OA. The dysregulation of the HIF-1α/CRAT/miR-144-3p signaling axis has a significant relationship with OA; silencing HIF-1α leads to downregulation of CRAT, which then results in increased expression of miR-144-3p causing dysfunction of peroxisomes and accumulation of long-chain fatty acids, promoting the development of OA (118).

**Table 3:** The role of HIF in SAS and OA.

Impact/Drug	Molecule/pathway	Function	
Hypoxia	HIF-1α(+)	It can be used as a biomarker of sleep apnea and is related to the overexpression of circadian clock proteins	Gabryelska et al. (105), Gabryelska et al. (106), and Liu et al. (104)
Hypoxia	NF-κB/HIF-1α (+)	Myoblast pyrosis	Yu et al. (47)
Hypoxia	ROS-HIF-1α-MMPs(+)	Aortic dissection	Liu et al. (108)
Hypoxia	ASC/HIF-1α(+)	Aggravated neuroinflammation and pyroptosis in early brain injury after subarachnoid hemorrhage	Xu et al. (109)
Hypoxia	HIF-1α(+),VEGF(+),Notch (+)	It can accelerate the angiogenesis of cartilage and regulate the autophagy and apoptosis of chondrocytes	Chen et al. (112) and Zeng et al. (113)
LncHIFCAR(−)	HIF-1α(+),VEGF(+),BNIP3(+),PI3K/AKT/mTOR (+)	It has a protective effect on chondrocytes	Sun et al. (114)
Casticin	HIF-1α/NLRP3 inflammasome(−)	Reduce osteoarthritis	Li et al. (115)
Agnuside	HIF-1α(−),NLRP3 inflammasome(−)	Reduces synovitis and fibrosis in experimental osteoarthritis	Zhang et al. (116)
Vitexin	HIF-1α(−)	It alleviates the inflammatory response of chondrocytes	Yang et al. (117)
HIF-1α(−)	HIF-1α(−),CRAT(−),miR-144-3p(+)	Peroxisome dysfunction and long-chain fatty acid accumulation	Song et al. (118)
CircRNA-UBE2G1(+)	HIF-1α(+)	It also increases the expression of pro-inflammatory cytokines and promotes the formation of osteoarthritis	Chen et al. (119)
miR-373(−)	HIF-1α(+)	It also increases the expression of pro-inflammatory cytokines and promotes the formation of osteoarthritis	Chen et al. (119)
Capsiate	SLC2A1(+),HIF-1α(−)	Slowing the progression of ferroptosis-related osteoarthritis	Guan et al. (120)
Mechanical stress	NF-κB(+),HIF-2α(+),MMP13(+),ADAMTs-4(+)	Aggravated cartilage degradation	Li et al. (121)
Osthole	NF-κB(−),HIF-2α(−),	The cartilage degradation was alleviated	Chern et al. (122)
Resveratrol	SIRT1(+),HIF-2α(−)	The progression of osteoarthritis was prevented	Li et al. (123)
D-mannose	HIF-2α(−)	Reduce the sensitivity of chondrocytes to ferroptosis	Zhou et al. (124)
HIF-2α	Zn -ZIP8-MTF1 (+)	It also increases the influx of Zn(2+) into chondrocytes and further promotes the expression of matrix-degrading enzymes	Lee et al. (125)
Syndecan-4(−)	miR-96-5p(+),HIF-2α(−)	Reduce cartilage degradation in osteoarthritis	Zhou et al. (126)
IkappaBalpha kinase inhibitor	NF-κB/HIF-2α (−)	Reduced cartilage degradation	Murahashi et al. (127)

* “+” represents facilitation, “−” represents inhibition.

Nonetheless, certain studies refute the protective role of HIF-1 in OA. For instance, in knee OA, elevated HIF-1α may exacerbate synovial fibrosis and fibroblast-like synoviocyte apoptosis (130). Moreover, LncHIFCAR has been shown to positively regulate HIF-1α and its target genes (including VEGF, BNIP3) along with the PI3K/AKT/mTOR pathway, thereby promoting chondrocyte apoptosis (114). Interestingly, Li et al. (115) discovered that Casticin mitigates knee OA through the suppression of HIF-1α/NLRP3 inflammasome signaling. In a similar vein, Agnuside alleviates synovitis and fibrosis in experimental knee OA by inhibiting the accumulation of HIF-1α and the activation of the NLRP3 inflammasome (116). Additionally, Vitexin targets the HIF-1α pathway to reduce inflammatory responses in osteoarthritic chondrocytes (117). Likewise, CircRNA-UBE2G1 modulates chondrocyte damage via the miR-373/HIF-1α pathway (119). Finally, Capsiate metabolites are found to reduce the progression of ferroptosis-associated OA by enhancing SLC2A1 expression, thereby effectively inhibiting HIF-1α expression (120).

HIF-2α is recognized as a crucial regulator of catabolic metabolism and the inflammatory cascade in OA (131). It can directly induce the expression of catabolic metabolic factors in chondrocytes and also enhance the expression of Fas in mature chondrocytes, thus mediating chondrocyte apoptosis and autophagy regulation (128). Moreover, mechanical stress has been found to exacerbate cartilage degradation through NF-κB and HIF-2α. Remarkably, inhibiting p65 has been shown to significantly reduce the expression of HIF-2α, thereby decreasing cartilage degradation and the production of related factors (121). Furthermore, HIF-2α is known to enhance the influx of Zn(2+) in chondrocytes by activating the Zn-ZIP8-MTF1 axis, consequently promoting the expression of matrix metalloproteinases and amplifying the regulatory effect on cartilage destruction in OA (125).

Suppressing HIF-2α offers a therapeutic strategy to relieve OA. Osthole has been demonstrated to alleviate cartilage degeneration by inhibiting the NF-κB and HIF-2α pathways (122). Intra-articular resveratrol injections activate SIRT1, thereby effectively inhibiting HIF-2α and preventing the progression of OA (123). Additionally, chemically modified curcumin targets the NF-κB/Hif-2α axis while stabilizing the extracellular matrix, showing potential in slowing the progression of OA (132). D-Mannose reduces the sensitivity of chondrocytes to ferroptosis by inhibiting HIF-2α, aiming to alleviate OA (124).

Exploring further, additional strategies targeting HIF-2α involve employing IkappaBalpha kinase inhibitors and syndecan-4 inhibitors to address associated pathways. The inhibition of syndecan-4 has been found to reduce cartilage degradation in osteoarthritic mouse models by downregulating HIF-2α via miR-96-5p (126). Similarly, IkappaBalpha kinase inhibitors are reported to reduce cartilage degradation in mouse models by downregulating the NF-κB/HIF-2α axis (127).

### IL-6 and IL-8

Cytokines IL-6 and IL-8 play a significant role in sleep apnea. Research has shown increased levels of IL-6, TNF-α, and IL-8 in the serum and plasma of SAS patients, which are positively correlated with disease severity, age, and BMI (133). Furthermore, a consistent conclusion from another study is that IL-6 levels are associated with both glucose metabolism and sleep apnea (134). This increase in IL-6 levels might be linked to the reduced oxygen consumption observed in sleep apnea patients (135). However, Kurt et al. (136) reported no significant variance in plasma IL-6 levels between the SAS group and a control group. As for the levels of IL-8, they are closely associated with obesity. Carpagnano et al. (137) observed a significant increase in the levels of IL-8, ICAM, and neutrophil percentage in induced saliva of obese sleep apnea patients, non-obese sleep apnea patients, and obese non-sleep apnea participants, compared to healthy individuals.

It remains contentious whether treating sleep apnea can influence the levels of IL-6 and IL-8. Previous findings suggest that adenotonsillectomy can improve clinical symptoms and signs, but has a minimal effect on TNF-α and IL-6 inflammatory levels (138). However, recent studies offer a different view, showing a decrease in circulating polymorphonuclear leukocyte levels of IL-6, IL-8, and β2-adrenergic receptor mRNA after adenotonsillectomy in children with obstructive SAS (139). One study found elevated IL-6 levels in patients with SAS, but Continuous positive airway pressure (CPAP) had no significant inhibitory effect on IL-6 levels in adults with SAS (140). In contrast, another study, by comparing untreated SAS patients and a control group, found that untreated SAS patients had significantly higher levels of IL-8 in peripheral blood than the control group. Moreover, this study suggests that nCPAP treatment can reduce hypoxia and the production of inflammatory mediators induced by SAS (141).

IL-6 and IL-8 are associated with OA. Senescent chondroprogenitor cells from the articular cartilage of knee OA patients contribute to the senescence-associated secretory phenotype by releasing IL-6 and IL-8 (142). Notably, IL-6 increases the production of inflammatory cytokines and expression of hypertrophic markers in primary mouse chondrocytes by activating JAK2/STAT3 (143). Blocking IL-6 can enhance Treg function and mitigate the progression of OA (144). Interestingly, IL-6 inhibits the spheroid size of osteophyte cells in OA by inducing apoptosis and reducing extracellular matrix molecules, highlighting its suppressive role in the regulation of pathological osteophyte formation (145).

Long non-coding RNA can influence the expression of IL-6 in OA. In OA patients, long non-coding RNA FER1L4 is downregulated, while IL-6 is upregulated. Importantly, the upregulation of FER1L4 can inhibit IL-6 expression in human chondrocytes (146).

Overexpression of IL-6 can exacerbate OA, and notably, PM2.5 can increase the production of IL-6 in human OA, worsening the condition (147). Moreover, Platelet response protein 2 promotes the production of IL-6 in OA synovial fibroblasts via the PI3K/AKT/NF-κB pathway (148).

Inhibiting IL-6 can alleviate OA. For instance, Tofacitinib relieves OA by upregulating miR-149-5p levels, thereby inhibiting the expression of JAK1/TNF-α/IL-6 (149). Similarly, Achyranthes root demonstrates anti-inflammatory and antioxidative treatment effects on OA and rheumatoid arthritis models by inhibiting the expression of IL-6 mediated matrix metalloproteinase-3 and -13 (150). Additionally, inhibition of the nuclear receptor RORα alleviates cartilage damage in OA by regulating the IL-6/STAT3 pathway (151). Furthermore, Genistein reduces obesity-induced OA in mice by inhibiting IL-6 and MMP-13 (152). Research has shown that fibroblast growth factor 10 delays the progression of OA by inhibiting the IL-6/JAK2/STAT3 signaling pathway, reducing synovial fibrosis (153). Physical therapy can also affect IL-6 expression in OA. Notably, rapid walking exercise significantly improves patients’ daily functioning and physical ability, while notably reducing TNF-α and IL-6 levels (154). Additionally, pulsed electromagnetic fields improve cartilage matrix, chondrocyte apoptosis, and autophagy by inhibiting TNF-α and IL-6 signaling (155). It is also suggested that IL-8 may be a risk factor for OA, with serum IL-8 levels being associated with aggravated knee joint symptoms (156). Moreover, research indicates that IL-8/Kc is highly responsive to mechanical, inflammatory, and metabolic stress (157).

## Discussion

**Figure 1:**
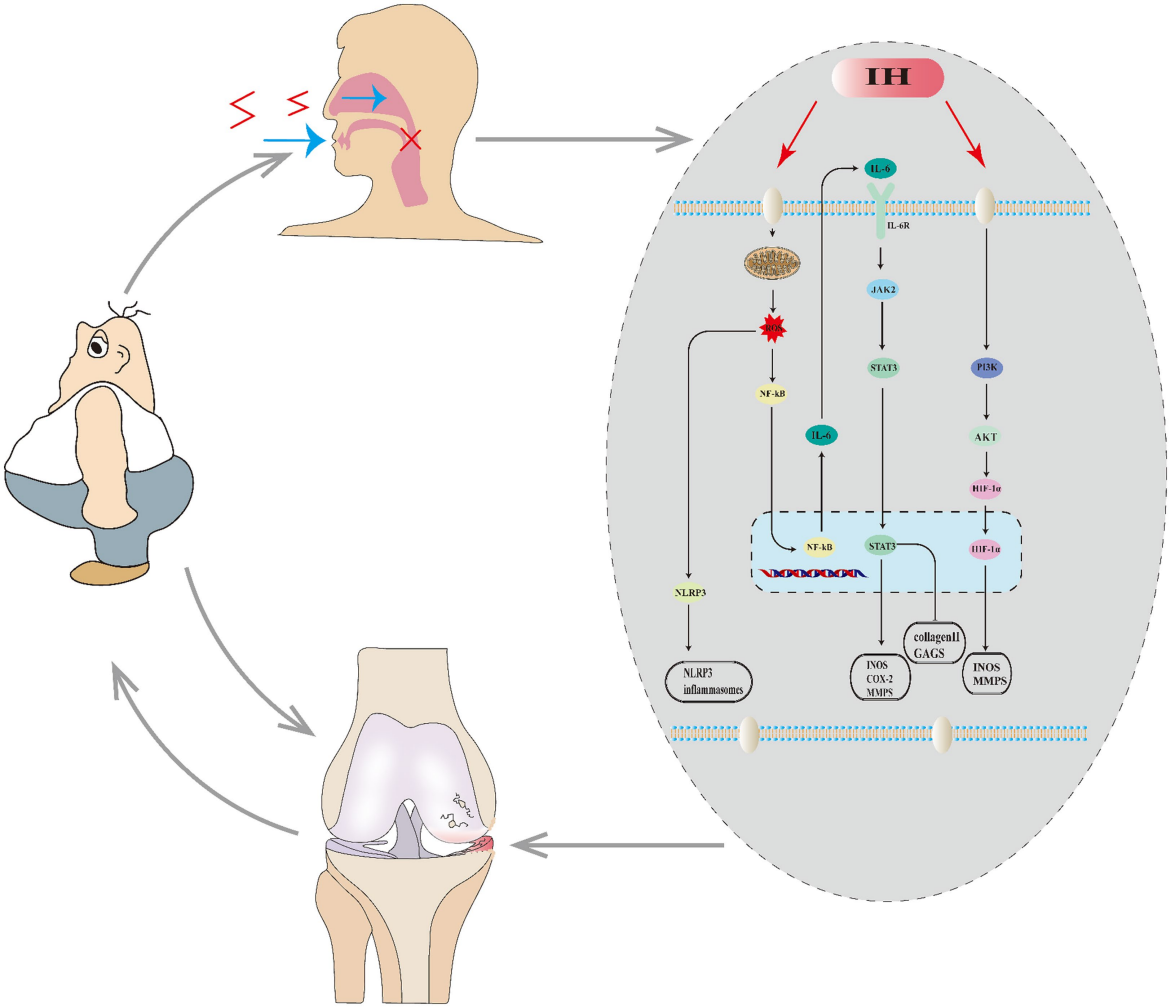
Relationship between OA and SAS. SAS causes intermittent hypoxia (IH) in the body, which affects a series of molecules and pathways, leading to the worsening of OA. OA, in turn, promotes obesity, which can further exacerbate both SAS and OA.

SAS and OA not only exhibit numerous common features, epidemiological studies reveal a tight correlation between them. Patients with both OA and SAS exhibit more severe symptoms of knee arthritis than those with only OA. Additional research has identified SAS as a risk factor for OA (3). There are numerous complex mechanisms involved (Figure 1). SAS results in intermittent hypoxia, which over time leads to mitochondrial dysfunction and significant ROS activation that stimulates inflammasomes (NLRP3), causing inflammation. ROS activates the NF-kB pathway, enhancing the production of inflammatory markers like IL-6 and TNF-α, which can expand the inflammatory response in joint synovium and lead to the breakdown of the extracellular matrix in cartilage cells, exacerbating OA.

Conversely, is OA a risk factor for SAS? Research in this area is still limited; however, Karla Diaz et al. (159) found a certain correlation between newly diagnosed SAS and prior OA. OA patients suffer from poor proprioception, reduced joint mobility, and weak muscle strength (160), leading to a loss of normal activity. A decrease in physical activity can result in becoming overweight and obese (161). Obesity further aggravates SAS. Overall, there is a bidirectional link between OA and SAS. SAS exacerbates OA, and decreased activity among OA sufferers could lead to obesity, thereby intensifying SAS. Owing to insufficient literature, it remains uncertain whether OA is a risk factor for SAS, but there appears to be a significant relationship between the two. Whether this relationship is causal or merely correlational needs more experimental evidence in the future.

In treating OA patients with SAS, intervention for SAS must not be neglected alongside OA treatment. Reducing weight is both essential and primary, and must be combined with correcting hypoventilation. CPAP is the gold standard for addressing hypoxia in SAS; however, its effectiveness on certain complications of SAS remains debated, potentially due to issues like patient compliance (162). Reduced patient compliance can result in poorer treatment effectiveness. Surgery to enhance hypoventilation is another method; Uvulopalatopharyngoplasty (UPPP) is the most frequently performed surgery for OSA. In traditional UPPP, tissue surrounding the blockage is removed to expand the airway and relieve obstruction (163). The success rate of the surgery is also related to the anatomical staging system; for stage IV patients, the success rate is only 20% (164). Excessive removal of obstructive parts and surrounding tissue to enlarge the airway space can cause complications such as nasopharyngeal narrowing and palatopharyngeal dysfunction (158). Hence, surgery is neither the preferred nor compulsory option, as its effectiveness in relieving small and multiple airway obstructions is limited.

For patients treating OA combined with SAS, relying solely on CPAP is insufficient; medication is also necessary (Tables 1–3), which targets and inhibits certain key molecules or pathways (Tables 1–3) to achieve therapeutic effects. Mitochondrial dysfunction is an upstream factor in these molecular pathways, and improving mitochondrial function might be a more effective method. In the future, treatment for patients with OA combined with SAS may involve improving or renewing mitochondrial function. Currently, a prevalent strategy includes the use of agents such as Urolithin A to boost mitochondrial functionality (165). Additionally, activating mitochondrial autophagy to clear damaged mitochondria can be achieved; for instance, artemisinin reduces TNFSF11 expression in cartilage and inhibits the PI3K/AKT/mTOR signaling pathway, thereby activating mitochondrial autophagy (166). Mitochondrial transplantation is another strategy; Lee et al. (167) have used mitochondrial transplantation to mitigate the progression of OA. Recently, liposomes have emerged as a new tool for transporting mitochondria (168), facilitating mitochondrial regeneration, representing a promising therapeutic strategy. Zhang et al. (169) have discovered that sustained release of melatonin can reactivate mitochondria in cartilage cells. Developing a sustained-release system for melatonin may also be a viable treatment method in the future.

## Conclusion

There is a close relationship between OA and SAS. SAS is a risk factor for OA, and OA might influence the progression of SAS, though the specifics of this influence require further experimental investigation. For patients with OA who have SAS, treating OA while also intervening timely in SAS is necessary, with weight reduction being a primary and essential step, alongside combined treatments such as CPAP and medications. Current drug development efforts involve numerous studies aimed at inhibiting or clearing molecular pathways, including ROS, NF-KB pathway, IL-6, and IL-8. Improving mitochondrial function may be an effective new method; renewing or transplanting mitochondria is a promising direction.
